# Time-Varying Associations of Physical Activity on Weekdays and Weekend Days in Minority Older Adults

**DOI:** 10.1093/geroni/igaa057.619

**Published:** 2020-12-16

**Authors:** Derek Hevel, Laurie Kennedy-Malone, Kourtney Sappenfield, Heidi Scheer, Christine Zecca, Jaclyn Maher

**Affiliations:** 1 University of North Carolina at Greensboro, Greensboro, North Carolina, United States; 2 UNCG School of Nursing, Greensboro, North Carolina, United States

## Abstract

Older adults are insufficiently physically active, and therefore, are at an increased health risk. However, less is known about the moment-to-moment physical activity behavior patterns that change across the day. The current study utilized accelerometry to better understand diurnal physical activity patterns. Minority older adults (N=91, age M=70.14, 96% Black/African American) participated in an 8-day study where they wore an ActivPAL accelerometer to measure physical activity. Physical activity was operationalized as stepping in the 60-minute window (±30-min) around a given moment. Time varying-effect modeling was used to determine how physical activity patterns change over the course of weekdays and weekend days. Results are rounded to the 5-min interval. On weekdays, time spent stepping increased from 8:00am (B=7.16, 95%CI: 5.04, 9.27) until peaking at 11:10am (B=8.46, 95%CI: 6.59, 10.33), slowly decreased to the lowest point at 6:10pm (B=6.72, 95%CI: 4.82, 8.62), and then increased slightly in the evening. On weekend days, time spent stepping was lowest at 8:00am (B=4.47, 95%CI: 2.14, 6.80), peaked at 12:20pm (B=5.50, 95%CI: 3.22, 7.78), gradually decreased until 5:50pm (B=5.21, 95%CI: 2.90, 7.52), and then increased slightly in the evening. Minority older adults engage in more stepping time around mid-day, but less stepping in the late afternoon to early evening on both weekdays and weekend days. Self-care activities (e.g., mealtime, bed-time routines) may influence older adults’ physical activity. This work identifies vulnerable times during the day when older adults engage in relatively low levels of physical activity which may be of interest for interventions.

